# When Adult Epiglottitis Does Not Improve With Intravenous Therapy: Think Epiglottic Abscess

**DOI:** 10.7759/cureus.108547

**Published:** 2026-05-09

**Authors:** Ahmed S Ahmed, Arshad Zubair, Mittal Sagar, Arun Cardozo

**Affiliations:** 1 Emergency Medicine, Lancashire Teaching Hospitals NHS Foundation Trust, Preston, GBR; 2 Otolaryngology - Head and Neck Surgery, Royal Preston Hospital, Preston, GBR; 3 Otolaryngology - Head and Neck Surgery, Lancashire Teaching Hospitals NHS Foundation Trust, Preston, GBR

**Keywords:** adult epiglottitis, airway management, ent pathology, epiglottic abscess, supraglottitis

## Abstract

Epiglottic abscess is a rare but potentially life-threatening complication of adult epiglottitis. Although airway compromise is a major concern, some patients may initially present with preserved airway patency despite significant supraglottic infection. We report the case of a 42-year-old woman who presented with a three-day history of worsening sore throat, dysphagia, odynophagia, fever, and inability to tolerate oral intake. Flexible nasendoscopy demonstrated marked supraglottic oedema and epiglottic enlargement without immediate airway compromise. Initial treatment with intravenous antibiotics, corticosteroids, analgesia, and supportive care resulted in minimal clinical improvement. Contrast-enhanced CT of the neck subsequently demonstrated a 3 cm rim-enhancing abscess within the epiglottis. The patient underwent microlaryngoscopy and operative drainage under general anaesthesia, with good postoperative recovery. This case highlights the importance of reconsidering the diagnosis in adult epiglottitis that fails to improve with medical therapy, even in the absence of overt airway obstruction. Early imaging and timely surgical intervention may be crucial in preventing further deterioration.

## Introduction

Adult epiglottitis remains an uncommon but serious infection of the upper airway and may still lead to significant morbidity despite current antibiotic treatment guidelines. Airway intervention is less common than in historical series, but the condition continues to demand careful assessment and close monitoring because deterioration may be sudden, and difficult airway management is well recognised. Epiglottic abscess is a rarer complication and may require escalation beyond conservative treatment, particularly when symptoms persist despite intravenous therapy [1-3].

Patients may present with severe sore throat, odynophagia, dysphagia, fever, muffled voice, drooling, or respiratory distress; however, the absence of stridor or obvious airway compromise does not exclude significant supraglottic pathology. Failure to improve with appropriate medical management should prompt reconsideration of the diagnosis and evaluation for complications such as abscess formation [2,4,5]. Flexible nasendoscopy is essential in diagnosing supraglottitis; however, marked inflammatory oedema may not reliably distinguish uncomplicated supraglottitis from an organised abscess cavity. In clinically stable patients with persistent symptoms despite intravenous therapy, contrast-enhanced CT may therefore help identify a drainable collection and exclude deeper extension.

## Case presentation

A 42-year-old woman presented to the emergency department with a three-day history of progressively worsening sore throat. She reported severe odynophagia and dysphagia and had been unable to tolerate food or fluids for the preceding 24 hours because of pain. She also described a two-day history of fever, nausea without vomiting, difficulty opening her mouth fully, drooling, difficulty speaking, and right-sided neck pain. Her general practitioner had prescribed oral penicillin prior to presentation, but she had been unable to tolerate oral medication. There was no shortness of breath or stridor on presentation.

Her past medical history included asthma, attention-deficit hyperactivity disorder, depression, anxiety, and previous tonsillitis. Records documented e-cigarette use. She had been taking methylphenidate for attention-deficit hyperactivity disorder and citalopram for depression/anxiety on a long-term basis.

On initial assessment, she appeared unwell and febrile, with a recorded temperature of 39.5°C. She was, however, able to speak in full sentences and maintained oxygen saturation on room air. Examination of the oropharynx demonstrated mildly inflamed tonsils without uvular deviation. There was tenderness over the right side of the neck with reduced neck movement. Flexible nasendoscopy demonstrated oedema of the supraglottis and arytenoids, with marked enlargement and thickening of the epiglottis. Vocal cord mobility was preserved, and there was no immediate airway compromise.

Initial blood investigations showed an inflammatory response. On admission, white blood cell count was 19.20 ×10⁹/L and C-reactive protein was 74 mg/L. Following admission under the otolaryngology team, she was managed with intravenous ceftriaxone and metronidazole, dexamethasone, analgesia, intravenous fluids, and close airway observation over the next 48 hours. After 48 hours of treatment, white blood cell count remained elevated at 15.35 ×10⁹/L, while C-reactive protein had fallen to 22.4 mg/L.

Despite 48 hours of treatment, she showed minimal clinical improvement. She continued to experience significant pain and ongoing dysphagia, with difficulty swallowing. Repeat ENT review and flexible nasendoscopy continued to demonstrate marked epiglottic swelling. Given the poor response to conservative treatment, further imaging was performed.

**Figure 1:**
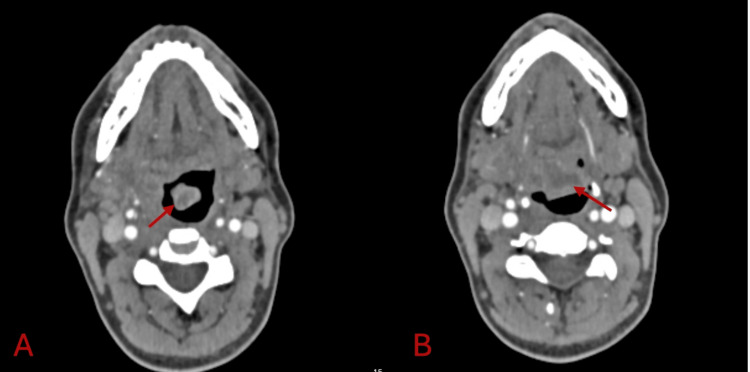
Axial contrast enhanced CT scan images of the neck. (A) Superior aspect of epiglottic abscess with arrow indicating the right aspect. (B) Inferior extent of the epiglottic abscess, extending to the right tongue base, with arrow indicating the main bulk of the abscess.

Contrast-enhanced CT of the neck demonstrated a rim-enhancing localised collection within the epiglottis involving both the suprahyoid and infrahyoid components, measuring approximately 3.0 cm in maximal axial dimension (Figure 1). The collection abutted the bilateral oropharyngeal walls and pharyngo-mucosal space. There was obliteration of the pre-epiglottic fat and associated inflammatory change extending into the right aryepiglottic fold. Reactive bilateral level II lymphadenopathy was also noted.

**Figure 2:**
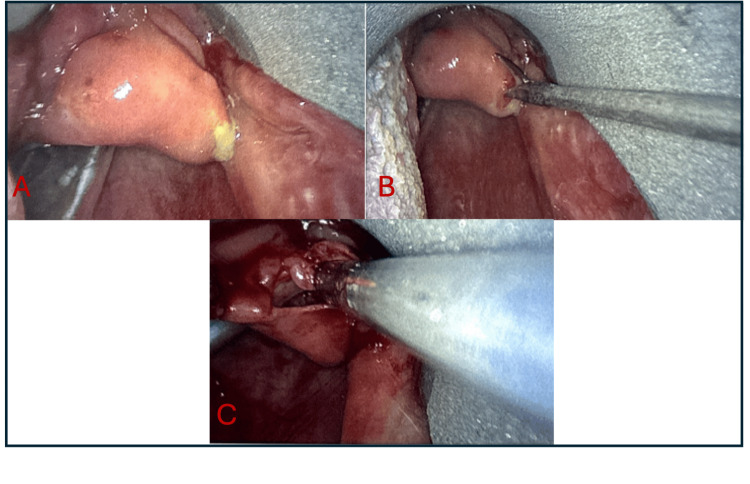
Intraoperative panel showing the epiglottic abscess during suspension microlaryngoscopy. (A) Intraoperative direct laryngoscopic image showing a swollen epiglottis, with pus extruding at the superior right aspect. (B) Incision created at the level of extrusion using micro-laryngoscopy, left-facing scissors. (C) Full evacuation of the abscess cavity with depth and extent displayed.

In view of the persistent symptoms, failure of intravenous therapy, and CT-confirmed abscess, the patient was taken to theatre for microlaryngoscopy and drainage of the epiglottic abscess under general anaesthesia. Following induction, the airway was secured with a size 5 microlaryngeal tube. A Lindholm laryngoscope was inserted and placed in suspension (Figure 2). The epiglottis was noted to be markedly swollen, with purulent material draining from the right side. The abscess was drained using microlaryngeal instruments. A swab of the purulent material was obtained, and the abscess cavity was marsupialised.

Postoperatively, the patient improved clinically. She was able to resume oral intake and reported improvement in throat pain, although some residual soreness initially persisted. She remained haemodynamically stable and was weaned from supplemental oxygen. Intravenous antibiotics were continued, and dexamethasone was tapered. Serial blood tests demonstrated improvement in inflammatory markers, particularly C-reactive protein, although leukocytosis persisted transiently. Repeat flexible nasendoscopy demonstrated resolution of the major supraglottic swelling. Intraoperative pus swab did not isolate any organisms, likely reflecting prior antibiotic exposure. She completed a total 14-day course of antibiotics, including intravenous ceftriaxone and metronidazole followed by oral co-amoxiclav on discharge. No recurrence of symptoms was identified during the three-month follow-up period.

## Discussion

This case illustrates several important features of adult epiglottitis complicated by epiglottic abscess. First, severe local disease may be present despite a preserved airway and the absence of stridor. Although large modern reviews suggest that most adults with epiglottitis do not require airway intervention, the condition remains high risk because deterioration can be abrupt and intubation may be challenging [1,3,6].

Second, failure to improve after appropriate intravenous treatment should trigger reassessment. In this case, the persistence of odynophagia and dysphagia despite 48 hours of antibiotics and steroids prompted repeat evaluation and CT imaging, which identified a 3-cm epiglottic abscess. This pattern has been described in previous reports of adult epiglottic abscess, where an apparently straightforward epiglottitis picture later proved to represent a drainable collection [4,7].

Third, CT was useful in this clinically stable patient because it confirmed the diagnosis, demonstrated the extent of the collection, and excluded deep neck space spread. Historically, epiglottic abscess has been reported in approximately 4% of adult supraglottitis cases; however, more recent series in which imaging has been used more routinely have reported higher rates, suggesting that this complication may be under-recognised in usual practice [5,8]. In adults who are stable enough to undergo imaging, CT can therefore be particularly valuable when symptoms are disproportionate, endoscopic findings are marked, or recovery is delayed [2,5,8].

Airway planning is also central in these cases. Even when the airway is initially patent, coordinated management between otolaryngology and anaesthetic teams is essential because the combination of supraglottic oedema, distorted anatomy, and a drainable collection can make airway intervention difficult if deterioration occurs [1,4]. In this case, operative drainage was undertaken under controlled conditions, allowing definitive management before the development of respiratory compromise.

Finally, the literature describes several drainage techniques for epiglottic abscess, including endoscopic cold-steel drainage, laser marsupialisation, and needle aspiration in selected cases [4,9,10]. Our patient was managed successfully with microlaryngoscopic drainage and marsupialisation. Overall, this case supports timely operative intervention when conservative treatment fails, rather than waiting for progressive airway compromise.

## Conclusions

Epiglottic abscess should be considered in adults with epiglottitis who fail to improve with intravenous therapy, even when the airway initially appears stable. Persistent dysphagia, odynophagia, and marked epiglottic swelling should prompt re-evaluation and, where feasible, contrast-enhanced imaging. Early recognition and timely operative drainage may prevent deterioration and lead to favourable recovery.
